# Levosimendan in Patients with Left Ventricular Dysfunction Undergoing Cardiac Surgery: An Update Meta-Analysis and Trial Sequential Analysis

**DOI:** 10.1155/2018/7563083

**Published:** 2018-05-08

**Authors:** Benji Wang, Xiaojie He, Yuqiang Gong, Bihuan Cheng

**Affiliations:** ^1^Department of Anesthesiology, Critical Care and Pain Medicine, The Second Affiliated Hospital and Yuying Children's Hospital of Wenzhou Medical University, Wenzhou, Zhejiang 325000, China; ^2^School of Ophthalmology and Optometry and Eye Hospital, Wenzhou Medical University, Wenzhou, Zhejiang 325000, China

## Abstract

**Background:**

Recent studies suggest that levosimendan does not provide mortality benefit in patients with low cardiac output syndrome undergoing cardiac surgery. These results conflict with previous findings. The aim of the current study is to assess whether levosimendan reduces postoperative mortality in patients with impaired left ventricular function (mean EF ≤ 40%) undergoing cardiac surgery.

**Methods:**

We conducted a comprehensive search of PubMed, EMBASE, and Cochrane Library Database through November 20, 2017. Inclusion criteria were random allocation to treatment with at least one group receiving levosimendan and another group receiving placebo or other treatments and cardiac surgery patients with a left ventricular ejection fraction of 40% or less. The primary endpoint was postoperative mortality. Secondary outcomes were cardiac index, pulmonary capillary wedge pressure (PCWP), length of intensive care unit (ICU) stay, postoperative atrial fibrillation, and postoperative renal replacement therapy. We performed trial sequential analysis (TSA) to evaluate the reliability of the primary endpoint.

**Results:**

Data from 2,152 patients in 15 randomized clinical trials were analyzed. Pooled results demonstrated a reduction in postoperative mortality in the levosimendan group [RR = 0.53, 95% CI (0.38–0.73), *I*^2^ = 0]. However, the result of TSA showed that the conclusion may be a false positive. Secondary outcomes demonstrated that PCWP, postoperative renal replacement therapy, and length of ICU stay were significantly reduced. Cardiac index was greater in the levosimendan group. No difference was found in the rate of postoperative atrial fibrillation.

**Conclusions:**

Levosimendan reduces the rate of death and other adverse outcomes in patients with low ejection fraction who were undergoing cardiac surgery, but results remain inconclusive. More large-volume randomized clinical trials (RCTs) are warranted.

## 1. Introduction

Cardiac surgery is a common operation, with more than 1 million procedures performed annually in the United States and Europe [1]. Though medical treatment and surgical techniques continue to improve, the management of patients undergoing cardiac surgery remains challenging. Postoperative risk of mortality remains high, especially in patients with postoperative low cardiac output syndrome. Preoperative left ventricular dysfunction is an independent risk factor for mortality and is also associated with postoperative low cardiac output syndrome [2]. This syndrome increases the risk of complications including the need for dialysis, stroke, and mechanical circulatory support [3–5]. Inotropic agents are used as first-line treatment to treat this syndrome. Unfortunately, most inotropic agents either give adverse reactions or impose unknown safety hazards [6]. Hence, new drugs with fewer deleterious effects are being sought.

Levosimendan is a calcium-sensitizing drug that increases cardiac contractility with vasodilatory properties [7] and does not impair diastolic relaxation [8]. And other therapeutic effects of levosimendan include reduction of oxidative burst activity of polymorphonuclear leucocytes (PMNs), immunomodulation, and antiapoptotic properties [9]. Several epidemiological studies suggest that levosimendan prevents low cardiac output syndrome and reduces postoperative mortality [10–13]. Therefore, in several countries, the drug was approved for the prevention and treatment of the low cardiac output syndrome following cardiac surgery [14–17]. However, recent large randomized clinical trials [18, 19] showed no survival benefit from levosimendan in patients with left ventricular dysfunction undergoing cardiac surgery.

Therefore, we systematically searched and analyzed randomized clinical trials (RCTs) to evaluate the effects of levosimendan in patients with left ventricular dysfunction undergoing cardiac surgery.

## 2. Methods

### 2.1. Literature Search Strategy

This systematic review and meta-analysis was performed following Preferred Reporting Items for Systematic reviews and Meta-Analysis guidelines [20]. Relevant studies investigating the perioperative use of levosimendan in patients undergoing cardiac surgery were independently searched in PubMed, EMBASE, and Cochrane Library Database and were last updated on November 20, 2017. There were no restrictions regarding languages, regions, or publication types. The search terms included “levosimendan”, “levosimedan”, “cardiac surgery”, “heart surgery”, and “randomized clinical trial”. The search strategy is shown in the Appendix. Additional eligible studies were identified by examination of the reference lists of the obtained publications and relevant reviews.

### 2.2. Study Selection

Two authors (Benji Wang and Xiaojie He) independently reviewed the titles, abstracts, or both and summarized the data from the selected articles. Any discrepancies in extracted data were resolved by the senior author (Bihuan Cheng). Studies were considered eligible for analysis if (1) patients were undergoing cardiac surgery with left ventricular dysfunction (left ventricular ejection fraction (EF) ⩽ 40%) [3], (2) the intervention was levosimendan, (3) the outcome of interest was mortality, including in-hospital or 30-day mortality, and (4) the study design was an RCT (i.e., not conference abstracts, case report, or review). Exclusion criteria were nonhuman experimental studies, pediatric studies, duplicate publications, and lack of mortality data.

### 2.3. Data Extraction

The following information was extracted: authors, year of publication, mean age of participants, number of participants, diagnostic criteria, preoperative mean EF, control treatment, levosimendan dose, and length of treatment (Table 1). Postoperative mortality was the primary endpoint. 30-day mortality was the first choice. If both in-hospital and 30-day outcomes were reported, the latter was used for analysis. The secondary endpoints were cardiac index, PCWP, length of ICU stay, postoperative atrial fibrillation, and postoperative renal replacement therapy.

### 2.4. Quality Assessment

Procedural and main outcomes were independently screened by two reviewers (Benji Wang and Xiaojie He), with divergences resolved by consensus. If consensus could not be reached, we consulted a third reviewer (Yuqiang Gong). Methodological quality evaluation was assessed according to the Cochrane Collaboration methods, judging risk of selection bias, performance bias, detection bias, attrition bias, and selective outcome reporting bias. We classified sources of bias as low, high, or unclear to indicate whether adequate measures were taken to protect against each potential source of bias [21].

### 2.5. Statistical Analysis

Binary outcomes in each study were expressed as relative risk (RR) with pertinent 95% confidence intervals (CIs). Weighted mean differences (WMDs) and 95% CIs were calculated for continuous variables. Studies that had no deaths in either group, that is, with no difference in the mortality rates, were discarded in the meta-analysis. Statistical heterogeneity was evaluated using Cochrane *Q* tests and *I*^2^. In general, *I*^2^ = (*Q* − df)/*Q*100%, where *Q* is the chi-squared statistic and df is the degrees of freedom, ranging from 0 to 100%. *I*^2^ > 50% suggested significant heterogeneity. The fixed-effects model was used if no substantial heterogeneity was observed; otherwise, the random-effects model was used. [22]. Publication bias was evaluated by inspection of the funnel plot. Analyses were performed with Review Manager 5.3 (Cochrane Collaboration, Oxford, UK). All statistical tests were 2-sided and *α* < 0.05 was considered to be significant [23].

When data were too sparse, we needed to judge the authenticity and reliability of the conclusions [24, 25]. TSA was similar to interim analyses in a single trial in which sequential monitoring boundaries were used [26, 27]. We conducted TSA assuming a 9% control event rate, 20% relative risk reduction, 90% power, and a two-sided 0.05 to determine the reliability of the primary endpoint [28, 29]. The sample size (optimal information size) was calculated. TSA software was from the Copenhagen Trial Unit (http://www.ctu.dk/tsa/).

## 3. Results

### 3.1. Literature Search

**Figure 1:**
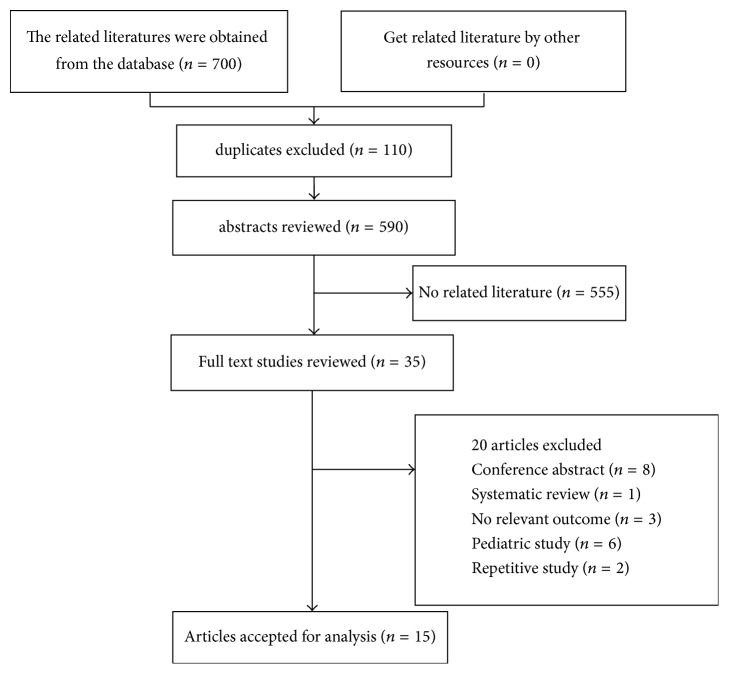
PRISMA flow diagram for trial selection.

According to the search strategy (Appendix), a total of 700 related studies were retrieved. After removing duplicate studies and excluding irrelevant titles or abstracts, 35 articles remained. After detailed examination, 15 RCTs (2,152 participants) were included in the final analysis [10, 11, 15–19, 30–37]. The flow chart summarizing the process of study selection is shown in Figure 1.

### 3.2. Study Characteristics

**Figure 2:**
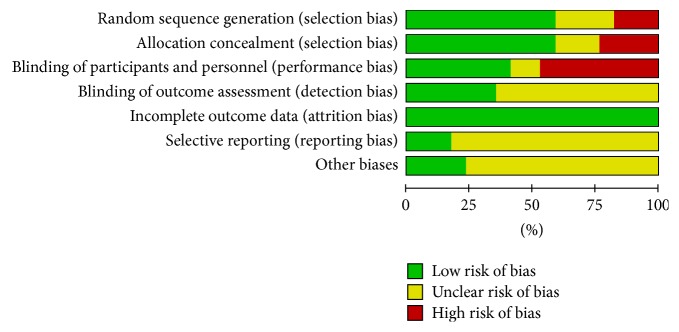
Methodological quality graph: review authors' judgements about each risk of bias item presented as percentages across all included studies.

**Figure 3:**
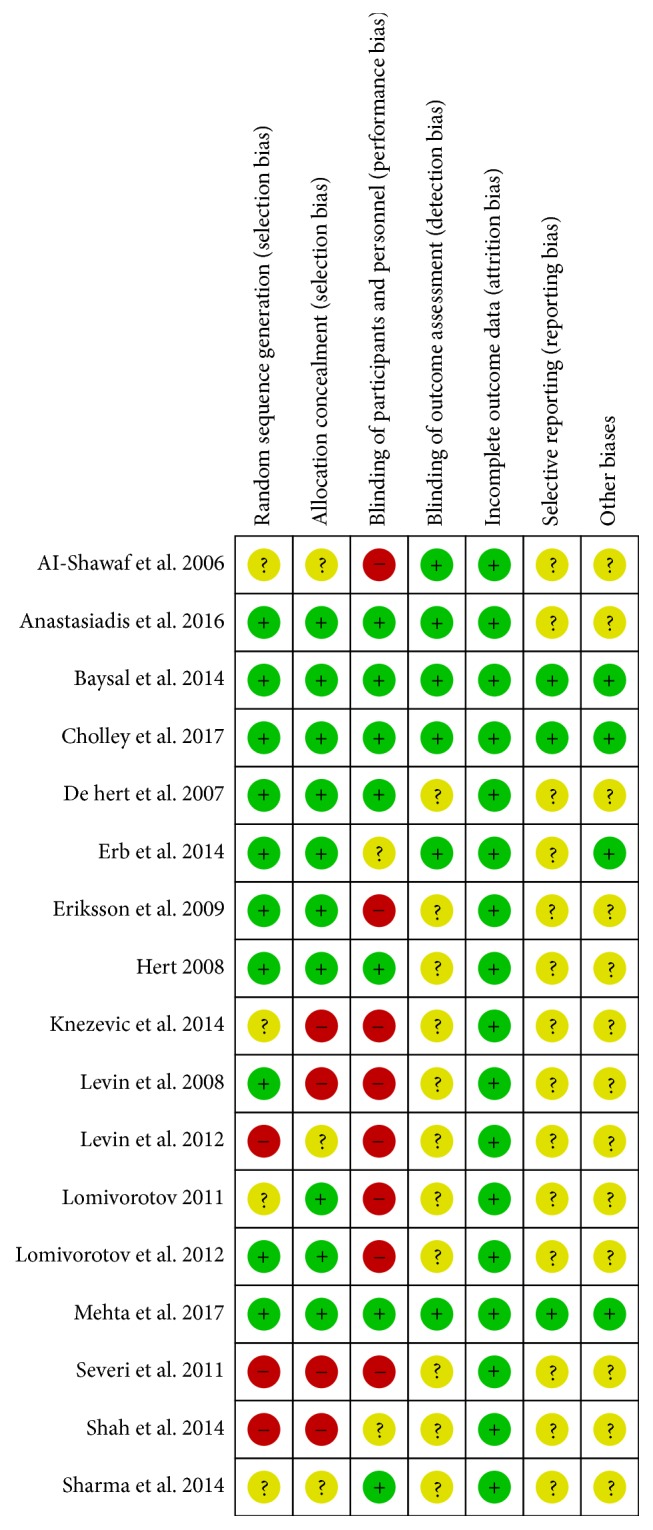
Methodological quality summary: review authors' judgements about each risk of bias item for each included study.

The principal features of the included studies are displayed in Table 1. Publication years range from 2006 to 2017. The surgical procedures included elective cardiac surgery with cardiopulmonary bypass (CPB) [10, 18, 36], elective coronary aortic bypass grafting (CABG) surgery [15, 17, 19, 30–34, 37], coronary surgery with extracorporeal circulation (ECC) [35], valve surgery [11, 31, 33], and heart transplantation [16]. All enrolled patients had a preoperative mean EF ⩽40%. Milrinone, dopamine, placebo, intra-aortic balloon pump (IABP), and standard inotropic agents were considered the control groups for comparison with levosimendan. We used the RCT quality evaluation standard described in the Cochrane Review Handbook. The assessments of the quality and risk of bias for each of the included studies are shown in Figures 2 and 3.

### 3.3. Quantitative Data Synthesis Analysis

**Figure 4:**
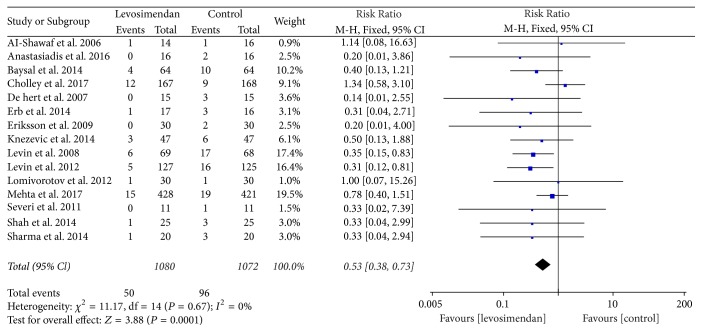
Forest plot for the effect of levosimendan on postoperative mortality.

For the primary endpoint, the pooled results from the fixed-effects model combining the risk ratio showed a significant reduction in the risk of death with levosimendan (Figure 4): 50 of 1080 patients in the levosimendan group and 96 of 1072 patients in the control group [RR = 0.53, 95% CI (0.38–0.73), *p* for heterogeneity = 0.67, *I*^2^ = 0].

Seven studies [11, 15, 17, 30, 32, 35, 37] reported cardiac index, which was significantly lower in the levosimendan group [RR = 0.66, 95% CI: (0.62, 0.70), *p* for effect < 0.00001]. There was also a significant reduction in PCWP [15, 30, 32–35, 37] [RR = −2.35, 95% CI: (−2.78, −1.93), *p* for effect < 0.00001], length of ICU stay [11, 17–19, 30–33, 36, 37] [RR = −0.48, 95% CI: (−0.72, −0.24), *p* for effect < 0.0001], and postoperative renal replacement therapy [10, 11, 15, 17, 18, 31, 32, 35] [RR = 0.51, 95% CI: (0.33, 0.77), *p* for effect = 0.002] in the levosimendan group. In addition, there was no difference in postoperative atrial fibrillation [10, 11, 15, 18, 19, 30–33, 35, 36] (RR = 0.97 [95% CI: 0.85, 1.09], *p* for effect = 0.60) (Table 2).

We conducted subgroup analyses by administration of levosimendan and by type of cardiac surgery (Table 3). Seven studies [10, 11, 18, 32, 34, 35, 37] taking bolus and 24-hour prolonged infusion of levosimendan suggested that there was a significant reduction in the risk of postoperative mortality in the levosimendan group (RR = 0.48 [95% CI: 0.32, 0.73], *p* for effect = 0.0004). Lacking the bolus or unclear duration did not suggest apparent difference. The subgroup analysis by type of cardiac surgery suggested that both coronary surgery and other surgical types in this analysis could lower the mortality in the levosimendan group (RR = 0.56 [95% CI: 0.35, 0.90], *p* for effect = 0.02 and RR = 0.50 [95% CI: 0.32, 0.78], *p* for effect = 0.002).

### 3.4. Risk of Bias and Sensitivity Analysis

**Figure 5:**
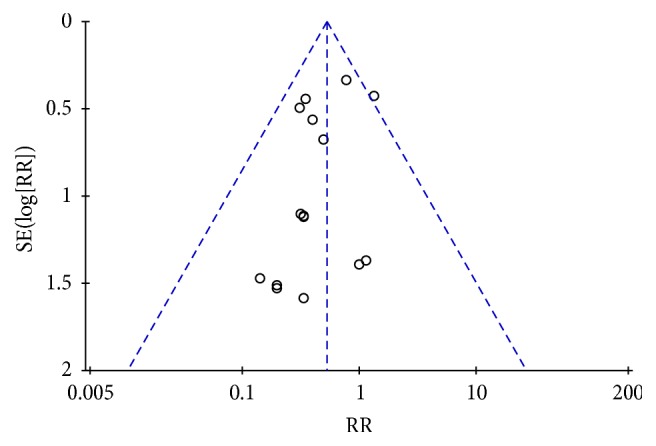
Funnel plot for the risk of levosimendan on postoperative mortality.

The funnel plot did not show substantial asymmetry with respect to estimate distribution. This suggests no small study bias regarding postoperative mortality (Figure 5). Sensitivity analyses were conducted to investigate the influence of single trials on overall risk estimates. The results did not substantially change following removal of any single study: 0.45 (95% CI: 0.31–0.64)–0.57 (95% CI: 0.40–0.81) for risk of postoperative mortality. This suggests that our results are statistically reliable.

### 3.5. Reliability Analysis of the Primary Endpoint

**Figure 6:**
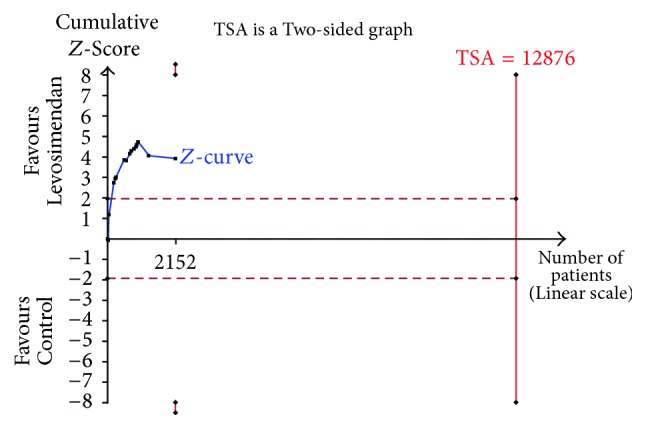
Trial sequential analysis of postoperative mortality on levosimendan compared with any control for low cardiac output syndrome in patients undergoing cardiac surgery.

We conducted TSA to determine the reliability of the primary outcome (Figure 6). TSA of levosimendan compared with control treatment indicated that the optimal information size needed to reliably detect a plausible effect was 12,876 patients. However, only 2,152 patients had so far been collected, far below optimal information size. The cumulative *z*-curve of all trials crossed the traditional boundary but did not cross the trial sequential monitoring boundary. These results suggest that the evidence may be false positive and unreliable.

## 4. Discussion

Our findings demonstrated that levosimendan treatment was associated with lower postoperative mortality compared with control treatment in patients with left ventricular dysfunction undergoing cardiac surgery. There was no clear evidence of between-trial heterogeneity. However, TSA suggested that the cumulative evidence might be false positive and unreliable. Additional trials are needed to confirm these conclusions. There was also a significant reduction in the rate of cardiac index, PCWP, length of ICU stay, and postoperative renal replacement therapy in the levosimendan group. No significant difference was observed in the incidence of postoperative atrial fibrillation. In addition, one approach of applying bolus and 24-hour prolonged infusion of levosimendan suggested that there was a significant reduction in the risk of postoperative mortality in the levosimendan group. These indicated that different dose and the duration of the infusion may lead to a different outcome. Furthermore, both coronary surgery and other surgical types could reduce the mortality in this analysis.

Previous meta-analyses [12, 13, 38, 39] showed a mortality benefit with levosimendan compared with other treatments in patients undergoing cardiac surgery. One of these meta-analyses demonstrated that levosimendan was associated with a greater effect among patients who had lower preoperative left ventricular systolic function compared with higher preoperative left ventricular systolic function [12]. These studies indicated that levosimendan played an important role in the treatment of postoperative low cardiac output syndrome, in high-risk patients undergoing cardiac surgery. However, two recent large trials [18, 19] showed inconsistent conclusions that levosimendan was not effective in reducing the incidence of postoperative mortality. Therefore, the aim of this study was to assess whether levosimendan infusion in patients with impaired left ventricular function (mean EF ⩽ 40%) who were undergoing cardiac surgery could reduce the postoperative mortality. Pooled evidence demonstrated that levosimendan was still associated with an increase in postoperative mortality. Moreover, the effects of levosimendan occur with decreasing postoperative PCWP, postoperative renal replacement therapy, length of ICU stay, and increasing cardiac index in analysis of secondary outcomes. These indicated that levosimendan was a treatment for postoperative low cardiac output syndrome (LCOS), in high-risk patients undergoing cardiac surgery.

Levosimendan has multiple potential mechanisms of action that may augment cardiac output with little increase in myocardial oxygen consumption [8]. Thus, levosimendan appears to be the ideal inotropic agent to support heart function in such patients [40]. One recent large trial [18] showed that levosimendan did not result in lower mortality compared with placebo, but it might help prevent LCOS and use of secondary inotrope. These data suggest that prophylactic levosimendan may have the potential to prolong survival among patients at risk for undergoing cardiac surgery.

We conducted the TSA to determine the reliability of the primary endpoint. Unfortunately, the result of TSA showed that the conclusion may be false positive and unreliable. Similarly, there was a significant reduction in the rate of postoperative atrial fibrillation with levosimendan in the previous meta-analysis [12]. However, updating the data and increasing the sample size, we saw no difference in postoperative atrial fibrillation. This explained that the conclusion was unreliable. Thus, an adequately powered trial assessing mortality reduction by levosimendan is needed. In addition, levosimendan is very expensive; formal recommendation of levosimendan requires evidence from cost-effectiveness studies [41].

The major advantage of this study was that we performed a rigorous screening of the literature and found high-quality literature. Mortality as a primary outcome is an important clinical outcome in critically ill patients. Furthermore, we conducted the TSA to assess the reliability and conclusiveness of the primary endpoint.

This study has some limitations. First, the sample of most studies in this analysis was small. Second, we included patients with preoperative mean EF ≤ 40%. However, the differences in EF of these studies were significant. The lowest mean EF was 17.56% [10]. This suggested that the severity of illness varied greatly and its prognosis also differed greatly [42]. Third, the dose and timing of levosimendan varied among trials. Some trials administered a loading dose, and we could not determine whether or not this variation affected results. Finally, the follow-up length of postoperative mortality varied; generally, 30-day mortality was the first choice. Several studies that only reported in-hospital mortality were included in this meta-analysis, possibly influencing the summary results.

## 5. Conclusions

In conclusion, levosimendan reduced the rate of death and other adverse outcomes in patients with low ejection fraction who were undergoing cardiac surgery. However, this result remains inconclusive, and more large-volume RCTs are warranted.

## Figures and Tables

**Table 1:** The main characteristics of included studies.

Study: first author	Year	Mean age of participants Levosimendan/control (years)	Number of participants	Setting	Preoperative mean EF (%)		Control	Levosimendan Infusion Dose (*μ*g/kg/min)
			Levosimendan/control		Levosimendan	Control		
Al-Shawaf et al.	2006	60.5/58	14/16	Elective CABG surgery, EF ≤ 35%	29 ± 6	31 ± 6	Milrinone	Bolus: 12 *μ*g/kg
								Inf: 0.1–0.2 *μ*g/kg/min
								Duration: 24 h

De hert et al.	2007	67/69	15/15	Elective cardiac surgery with CPB, EF ≤ 30%	24 ± 6	27 ± 3	Milrinone	Bolus: none
								Inf: 0.1 *μ*g/kg/min
								Duration: NR

Levin et al.	2008	62.4/61.7	69/68	Coronary surgery with ECC and manifest LCOS	36.62 ± 4.36	38.22 ± 5.24	Dobutamine	Bolus: 10 *μ*g/kg
								Inf: 0.1 *μ*g/kg/min
								Duration: 24 h

Eriksson et al.	2009	64/64	30/30	CABG with CPB and EF < 50% or acute CHF	36 ± 8	36 ± 8	Placebo	Bolus: 12 *μ*g/kg
								Inf: 0.2 *μ*g/kg/min
								Duration: 24 h

Severi et al.	2011	66/60	11/11	CABG with or without concomitant mitral surgery, EF < 50%	26 ± 6.2	30 ± 6.4	IABP	Bolus: none
								Inf: 0.1 *μ*g/kg/min
								Duration: 24 h

Lomivorotov et al.	2012	57.3/56.8	30/30	CABG with CPB, EF < 35%	31 (28–33)	30 (29–33)	IABP	Bolus: 12 *μ*g/kg
								Inf: 0.1 *μ*g/kg/min
								Duration: 24 h

Levin et al.	2012	63.7/62.9	127/125	Cardiac surgery with CPB, EF < 25%	17.56 ± 3.24	18.62 ± 2.12	Placebo	Bolus: 10 *μ*g/kg
								Inf: 0.1 *μ*g/kg/min
								Duration: 24 h

Sharma et al.	2014	53.95/54.55	20/20	CABG and mitral valve repair, EF < 25%	23.55 ± 4.87	22.55 ± 0.92	Placebo	Bolus: none
								Inf: 200 *μ*g/kg
								Duration: 24 h

Baysal et al.	2014	56.73/58.41	64/64	Mitral valve surgery, EF ≤ 45%	35.0 (20–50)	37.5 (25–50)	Standard inotropic agents	Bolus: 6 *μ*g/kg
								Inf: 0.1 *μ*g/kg/min
								Duration: 24 h

Erb et al.	2014	69.5/63.4	17/16	Elective CABG with or without valve surgery, EF ≤ 35%	22.0 ± 4.5	22.4 ± 5.5	Placebo	Bolus: none
								Inf: 0.1 *μ*g/kg/min
								Duration: NR

Knezevic et al.	2014	53/49	47/47	Heart transplantation and advanced heart failure	20 ± 6	21 ± 7	Standard inotropic or vasopressor	Bolus: none
								Inf: 0.1 *μ*g/kg/min
								Duration: NR

Shah et al.	2014	59.91/61.32	25/25	OPCABG, EF < 30%	22.45 ± 4.06	22.56 ± 3.41	Placebo	Bolus: none
								Inf: 0.13 *μ*g/kg/min
								Duration: 24 h

Anastasiadis et al.	2016	61.1/62.2	16/16	CABG, EF ≤ 40%	35.7 ± 4.9	37.5 ± 3.4	Placebo	Bolus: none
								Inf: 0.1 *μ*g/kg/min
								Duration: 24 h

Mehta et al.	2017	65/65	428/421	Cardiac surgery with CPB, EF ≤ 35%	26 (24–32)	27 (22–31)	Placebo	Bolus: 0.2 *μ*g/kg/min
								Inf: 0.1 *μ*g/kg/min
								Duration: 24 h

Cholley et al.	2017	69/67	167/168	CABG with CPB or combined with valve surgery, EF ≤ 40	≤40	≤40	Placebo	Bolus: none
								Inf: 0.1 *μ*g/kg/min
								Duration: 24 h

CABG: coronary aortic bypass grafting; OPCABG: off-pump coronary aortic bypass grafting; CBP: cardiopulmonary bypass; EF: ejection fraction; ECC: extracorporeal circulation; LCOS: low cardiac output syndrome; CHF: congestive heart failure; IABP: intra-aortic balloon pump.

**Table 2:** Secondary endpoints after randomizations.

Secondary outcomes	Number of studies	95% CI	*P* (heterogeneity)	*I* ^2^ (%)	*P* (overall effect)
Cardiac index (L/min/m2)	7	0.66 [0.62, 0.70]	<0.00001	89	<0.00001
Pulmonary capillary wedge pressure (mmHg)	7	−2.35 [−2.78, −1.93]	0.001	73	<0.00001
Postoperative atrial fibrillation	11	0.97 [0.85, 1.09]	0.0006	68	0.60
Postoperative renal replacement therapy	8	0.51 [0.33, 0.77]	0.86	0	0.002
Length of ICU stay (days)	10	−0.48 [−0.72, −0.24]	<0.00001	85	<0.0001

**Table 3:** Stratified analyses of levosimendan administration and surgical type.

Group	Number of studies	95% CI	*P* (heterogeneity)	*I* ^2^ (%)	*P* (overall effect)
Timing and dose of infusion of levosimendan					
Bolus + 24-hour prolonged infusion	7	0.48 [0.32, 0.73]	0.62	0	0.0004
No bolus + 24-hour prolonged infusion	5	0.79 [0.41, 1.53]	0.43	0	0.49
No bolus + unclear duration	3	0.35 [0.13, 1.00]	0.72	0	0.05
Type of cardiac surgery					
Coronary surgery	10	0.56 [0.35, 0.90]	0.58	0	0.02
Other cardiac surgeries	5	0.50 [0.32, 0.78]	0.47	0	0.002

## Appendix

### Searching Strategy

*PubMed.* Search (((((((((((((((Procedures, Cardiac Surgical[Title/Abstract]) OR Surgical Procedure, Cardiac[Title/Abstract]) OR Surgical Procedures, Cardiac[Title/Abstract]) OR Surgical Procedures, Heart[Title/Abstract]) OR Cardiac Surgical Procedure[Title/Abstract]) OR Heart Surgical Procedures[Title/Abstract]) OR Procedure, Heart Surgical[Title/Abstract]) OR Procedures, Heart Surgical[Title/Abstract]) OR Surgical Procedure, Heart[Title/Abstract]) OR Heart Surgical Procedure[Title/Abstract])) OR Procedure, Cardiac Surgical[Title/Abstract]) OR “Cardiac Surgical Procedures”[Mesh])) AND (((((((“simendan” [Supplementary Concept]) OR levosimendan[Title/Abstract]) OR levosimedan[Title/Abstract]) OR or 1259[Title/Abstract]) OR “or 1259”[Title/Abstract]) OR cardiotonic agent[Title/Abstract]) OR inodilator^*∗*^[Title/Abstract])) AND random^*∗*^[tw].

*Embase*

  #20#17 AND #18 AND #19
  #19#8 OR #9 OR #10 OR #11 OR #12 OR #13 OR #14 OR #15 OR #16
  #18#1 OR #2 OR #3 OR #4 OR #5 OR #6 OR #7
  #17random^*∗*^:ab,ti
  #16‘cardiac surgery':ab,ti
  #15‘surgical procedure, cardiac':ab,ti
  #14‘surgical procedures, cardiac':ab,ti
  #13‘surgical procedures, heart':ab,ti
  #12‘cardiac surgical procedure':ab,ti
  #11‘heart surgical procedures':ab,ti
  #10‘surgical procedure, heart':ab,ti
  #9‘heart surgical procedure':ab,ti
  #8‘heart surgery'/exp
  #7inodilator^*∗*^:ab,ti
  #6‘cardiotonic agent':ab,ti
  #5simdax:ab,ti
  #4‘or 1259':ab,ti
  #3levosimedan:ab,ti
  #2levosimendan:ab,ti
  #1‘levosimendan'/exp

*Cochrane*

  #1MeSH descriptor: [Thoracic Surgery] explode all trees
  #2CARDIAC SURGICAL PROCEDURES explode all trees (MeSH)
  #3 ((coronary next artery next bypass next surgery) or (coronary next artery next surgery) or (coronary next bypass next graft next surgery) or (coronary next artery next bypass next graft) or (coronary next bypass next graft) or (coronary next artery next bypass next graft^*∗*^) or (coronary next bypass next graft^*∗*^) or cabg or (((off next pump) or offpump or off-pump) and (coronary next surgery)) or (open next heart next surgery) or (heart next surgery) or (heart next valve next surgery) or (cardiopulmonary next bypass))
  #4CARDIOPULMONARY BYPASS explode all trees (MeSH)
  #5levosimendan or levosimedan or or1259 or “or 1259” or simdax
  #6(inotropic near/2 (agent^*∗*^ or drug^*∗*^ or medicat^*∗*^ or act^*∗*^))
  #7Einodilator^*∗*^
  #8(calcium near/2 sensiti^*∗*^)
  #9MeSH descriptor: [Cardiotonic Agents] this term only
  #10#1 or #2 or #3 or #4
  #11#5 or #6 or #7 or #8 or #9
  #12#10 and #11
